# Ischemic-hypoxic preconditioning enhances the mitochondrial function recovery of transplanted olfactory mucosa mesenchymal stem cells via miR-181a signaling in ischemic stroke

**DOI:** 10.18632/aging.202807

**Published:** 2021-04-04

**Authors:** Yi Zhuo, Wei Chen, Wenshui Li, Yan Huang, Da Duan, Lite Ge, Jialin He, Jianyang Liu, Zhiping Hu, Ming Lu

**Affiliations:** 1 The National and Local Joint Engineering Laboratory of Animal Peptide Drug Development, College of Life Sciences, Hunan Normal University, Changsha 410006, Hunan, P.R. China; 2 Hunan Provincical Key Laboratory of Neurorestoratology, The Second Affiliated Hospital Hunan Normal University, Changsha 410003, Hunan, P.R. China; 3 Department of Neurology, The Second Xiangya Hospital, Central South University, Changsha 410008, Hunan, P.R. China

**Keywords:** olfactory mucosa, mesenchyma stem cells, ischemic-hypoxic, miR-181a, stroke

## Abstract

Cerebral ischemia/reperfusion injury causes a series of intricate cascade reactions in brain tissue causing apoptosis and proinflammatory programmed cell death known as pyroptosis of nerve cells. The dysfunction of target organelle mitochondria plays a key role in the process of neuronal apoptosis and pyroptosis. Mesenchymal stem cells (MSCs) have been widely used in the experimental or clinical treatment of various ischemic diseases, but the therapeutic efficacy of MSCs on cerebral ischemia-reperfusion injury need to be improved. We successfully cultured olfactory mucosa MSCs (OM-MSCs) to obtain a better source of seed cells. In this way, the therapeutic potential of OM-MSCs transplantation has been evaluated for ischemic stroke using an optimized culture scheme *in vitro*. Ischemic-hypoxic preconditioned OM-MSCs (IhOM-MSCs) were used to treat a neuron model of oxygen-glucose deprivation/reperfusion and the middle cerebral artery occlusion in rats. These results demonstrated that IhOM-MSCs mediated the upregulation of the downstream target genes GRP78 and Bcl-2 by miR-181a to protect mitochondrial function and inhibit apoptosis and pyroptosis of neurons in the ischemia/reperfusion injury model. Thus, IhOM-MSCs transplantation may be an effective therapy of ischemic stroke in the future.

## INTRODUCTION

The most common cause of ischemic stroke is the embolization of arteries after a thrombus on the inner wall of a blood supply vessel falls off [1]. The brain is very sensitive to ischemia. After cerebral ischemia reperfusion injury, the body produces a wide range of responses, either actively or passively, which constitute the complicated pathological mechanism of cerebral ischemia/reperfusion injury [2, 3]. Mitochondrial dysfunction plays a key role in this process [4]. Mitochondrial permeability transition (MPT) is the central hub of mitochondrial internal and external information exchange, and plays a key role in maintaining mitochondrial function. The MPT uses a protein complex formed in the inner mitochondrial membrane, which changes under certain pathological conditions such as ischemic stroke. This may be accompanied by mitochondrial membrane potential (MMP) disorder and a series of reactions caused by a large amount of reactive oxygen species (ROS) [5, 6]. Cerebral ischemia/reperfusion injury is often associated with a severe neuroinflammatory response leading to different subtypes of programmed neuronal death, including apoptosis and pyroptosis [7]. Pyroptosis is a type of programmed cell death associated with inflammatory responses. Pyroptosis has similar characteristics but different mechanisms compared to apoptosis. It is not regulated by the apoptosis-related protein caspase-3, but depends on the regulation of the inflammation-related protein, caspase-1 [7]. The NLRP3 inflammasome, a macromolecular signaling complex, plays an important role in apoptosis. Cerebral ischemia/reperfusion injury can activate the NLPR3 inflammasome and promote the secretion of related inflammatory factors such as interleukin-1 (IL-1) and interleukin-18 (IL-18), thus inducing neuronal cell death [8].

Mesenchymal stem cells (MSCs) are widely used in the experimental or clinical treatment of various ischemic diseases, but the therapeutic effects of MSCs in ischemic stroke need to be determined [9, 10]. Therefore, to achieve better therapeutic effects for the treatment of ischemic stroke, optimizing MSC culture conditions, transplantation pathways and cell numbers is of the utmost importance.

To obtain a better source of seed cells for the treatment of central nervous system injury diseases, a culture system of olfactory mucosa MSCs (OM-MSCs) was successfully established [11]. OM-MSCs have the advantage of a superior capacity for proliferation, feasibility for autologous transplantation, convenient isolation, low immune rejection, high safety, no genetic mutations after infinite passaging *in vitro*, and are associated with fewer ethical issues [12–14]. We previously demonstrated that hypoxic pretreated OM-MSCs were specifically induced and differentiated into neurons and dopaminergic neurons, showing the therapeutic potential of treating central nervous system injuries such as Parkinson's disease [15]. OM-MSCs are a suitable seed cell source for cerebral diseases but the therapeutic effectiveness in ischemic stroke has not been evaluated.

The lesions associated with ischemic stroke are composed of a central area, or necrotic area, of severe ischemia and an ischemic penumbra, which refers to the surrounding area of the lesion transitioning to normal brain tissue. The penumbra is the area where energy metabolism is preserved and blood supply is inhibited [16, 17]. Studies have shown that miR-181a is significantly downregulated in the penumbra of a stroke model while having protective effects on neurons and glial cells [16]. We hypothesized that OM-MSCs could be pretreated with ischemia-hypoxia (IhOM-MSCs) by simulating the microenvironment in the penumbra of ischemic stroke. In this way, cells can adapt to the microenvironment of ischemia/hypoxia in advance. Subsequently, the downregulation of miR-181a in cells can increase the protective function of neuronal mitochondria and inhibit apoptosis and pyroptosis of neurons. This hypothesis was tested here in order to observe the effect of IhOM-MSC intervention on the oxygen-glucose deprivation/reperfusion (OGD/R) and middle cerebral artery occlusion (MCAO) models so that a possible mechanism could be elucidated. The results of the current investigation may provide a novel therapeutic approach to clinical ischemic stroke.

## RESULTS

### Identification and biological characteristics of OM-MSCs

**Figure 1:**
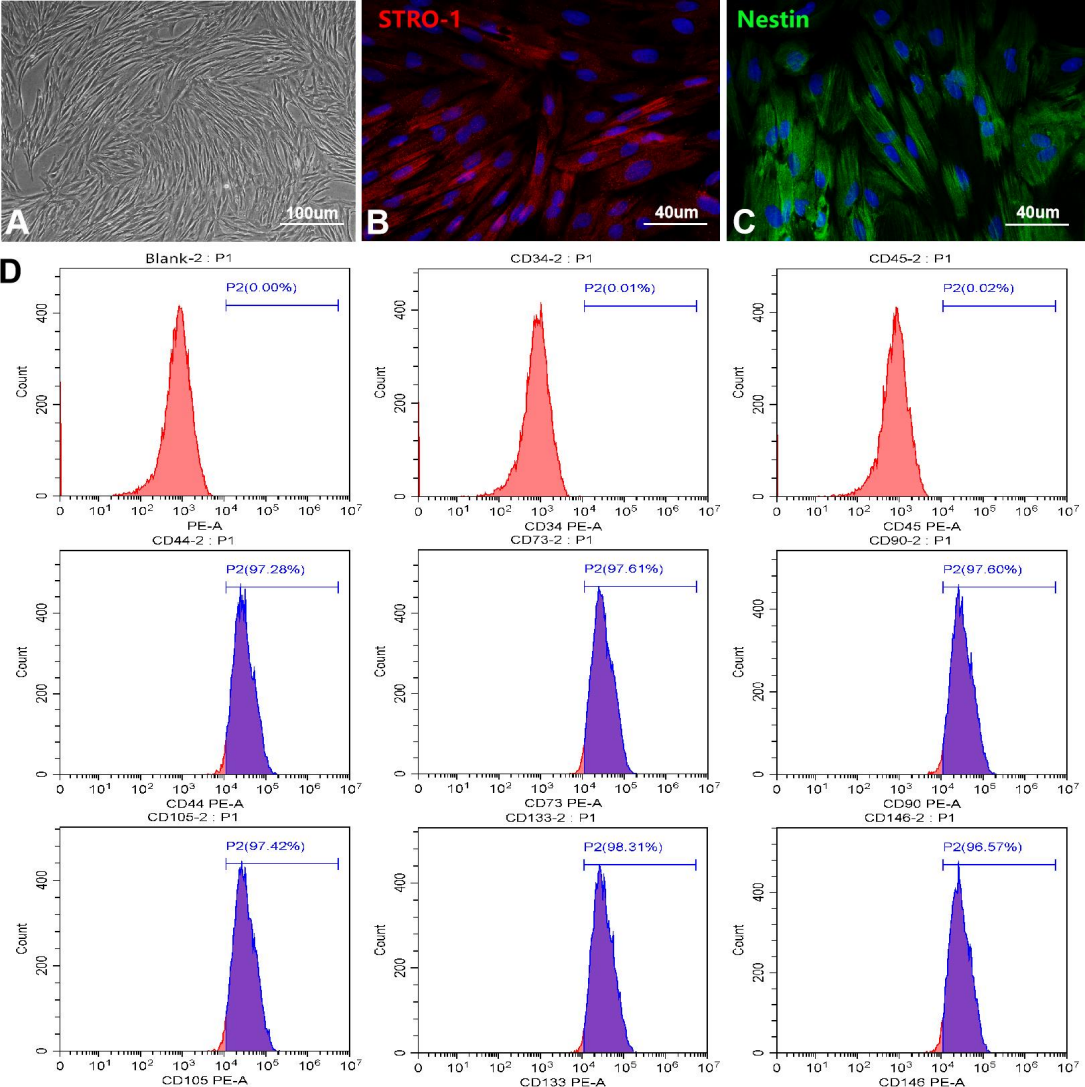
**Culture and identification of OM-MSCs.** (**A**) OM-MSCs were mainly exhibited spindle-shaped and a radial arrangement under light microscope. (**B**, **C**) The specific markers STRO-1 and Nestin of OM-MSCs were identified by immunofluorescence. (**D**) The surface markers and purity of OM-MSCs were detected by flow cytometry assay.

To find a more ideal source of seed cells for the treatment of central nervous system injury diseases, we successfully established a culture system of OM-MSCs. OM-MSCs exhibited a spindle-shape with a radial arrangement as observed using a light microscope (Figure 1A). Immunofluorescence revealed the intracellular localization of the stromal cell marker, STRO-1, as well as the stem cell marker, Nestin (Figure 1B, 1C). Flow cytometry assays revealed OM-MSCs highly expressed CD44, CD73, CD90, CD105, CD133, and CD146, but did not express CD34 and CD45, with an OM-MSC purity of over 97% (Figure 1D). Using the scanning electron microscope, there were many microvilli on the surface of the OM-MSCs (Supplementary Figure 1A). Observations of cells by transmission electron microscopy (TEM), revealed that OM-MSCs were in a relatively active phase (Supplementary Figure 1B). This was shown by two nuclei present in the same cell; both were round or oval in shape with large and obvious nucleoli. In addition, few organelles such as rough endoplasmic reticulum and mitochondria were found. As observed in the TEM, OM-MSCs were induced by all-trans retinoic acid and differentiated into neuron-like cells (Supplementary Figure 1C). The cells induced by a lipogenic inducer were stained with Oil Red O, which showed red lipid droplets in the cytoplasm (Supplementary Figure 1D). After induction by an osteogenic inducer, Alizarin Red staining showed red mineralized nodules in the cells (Supplementary Figure 1E). Finally, the cell cycle detection results showed that most of the OM-MSCs were in the quiescent state, while a small part of them were in the replication stage, and still retained the ability of cell renewal and proliferation, which was consistent with the growth characteristics of stem cells (Supplementary Figure 1F).

### Determination of the optimal cultivation scheme for IhOM-MSCs

**Figure 2:**
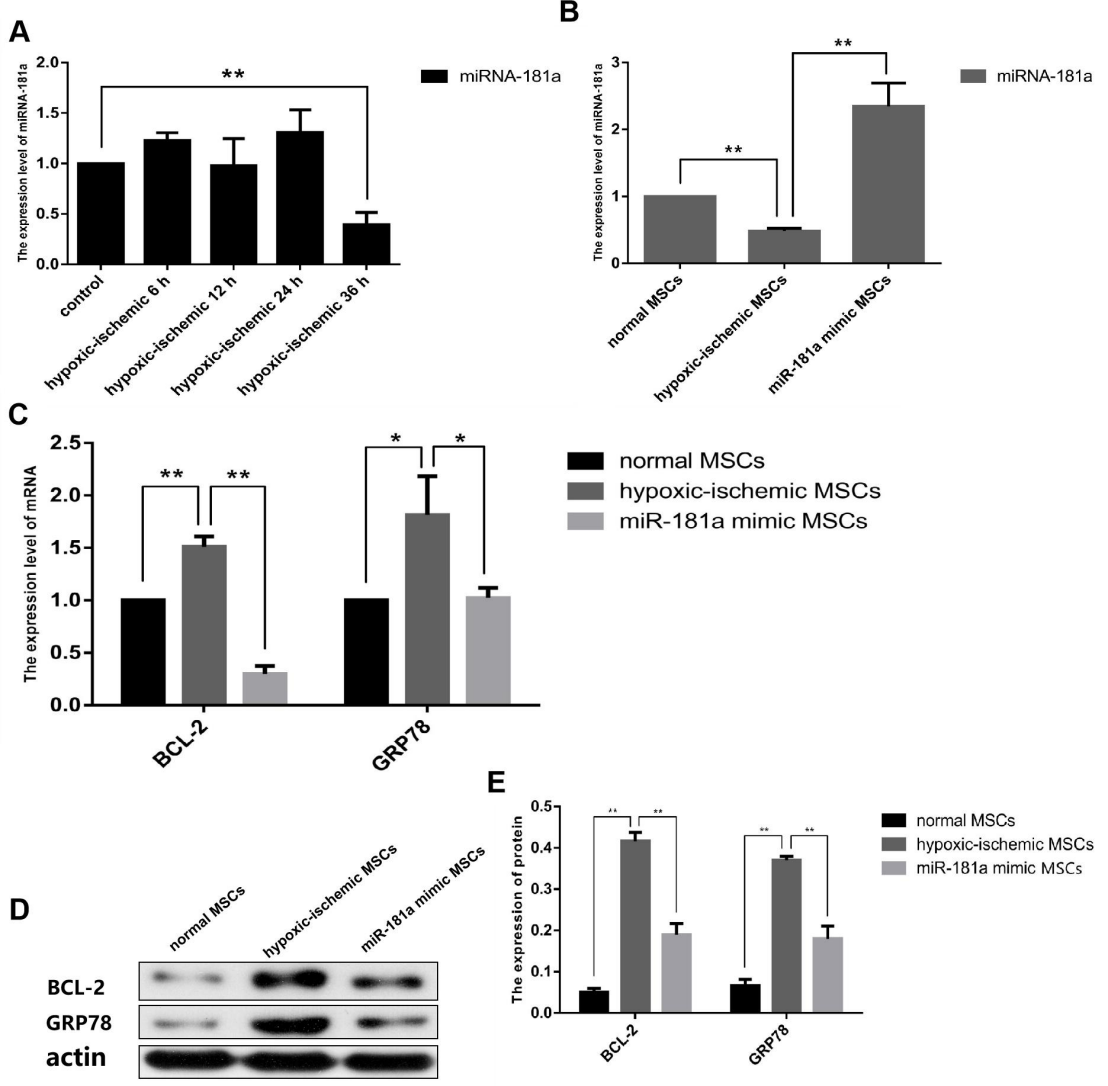
**Determination of optimal cultivation scheme of IhOM-MSCs.** (**A**) We were pretreated OM-MSCs with ischemia/hypoxia at different time gradients, and the RT-qPCR result showed that miR-181a was significantly down-regulated after 36h. (**B**) Successful mimic of miR-181a in IhOM-MSCs was verified by qPCR. (**C**–**E**) Protein and mRNA expression of GRP78 and Bcl-2 in OM-MSCs as determined by western blot and qPCR, respectively. (OM-MSCs were replaced by MSCs in the figure. All data are presented as the mean value ± SD. *p<0.05, **p<0.01).

According to the conclusion of our previous study, the serum concentration of culture medium was changed from 10% FBS to 5% FBS, and the oxygen concentration was changed to 3% O_2_. We first pretreated OM-MSCs with ischemia/hypoxia at different time gradients, and the experimental results showed that miR-181a was significantly downregulated after 36 h (Figure 2A). It was then important to confirm that the significant downregulation of miR-181a was caused by ischemic/hypoxia preconditioning. To do this, the expression of miR-181a was determined by OM-MSCs pre-transfected with an miR-181a mimic. It was found that miR-181a was not downregulated without pretreatment of the OM-MSCs (Figure 2B). Then, mRNA and protein expressions of downstream target genes, GRP78 and Bcl-2, of miR-181a in each group were detected by RT-qPCR and western blot analysis. The results showed that GRP78 and Bcl-2 were significantly upregulated in the IhOM-MSCs group relative to cells without pretreatment (Figure 2C–2E). Subsequently, immunofluorescence and flow cytometric phenotype markers were detected for IhOM-MSCs, which confirmed that the phenotype of IhOM-MSCs did not change relative to cells without pretreatment (Supplementary Figure 2B–2D). Finally, the flow cytometry detection of IhOM-MSCs and OM-MSCs showed a low apoptosis rate and no significant difference (Supplementary Figure 2E).

### Co-culture of IhOM-MSCs with SH-SY5Y neurons attenuates apoptotic cell death and pyroptotic cell death during OGD/R insult

**Figure 3:**
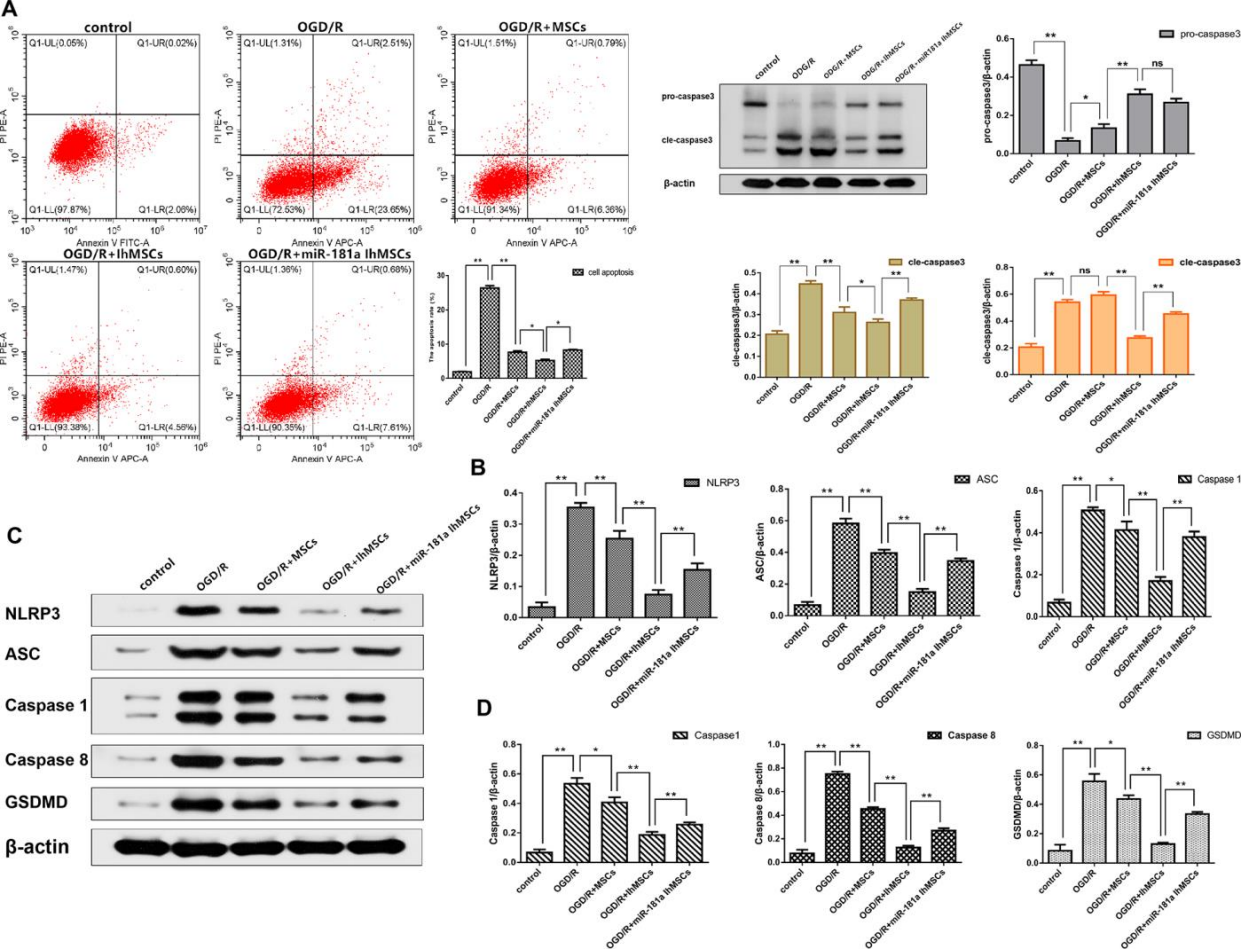
**NEW-Co-culture of IhOM-MSCs with SH-SY5Y neurons attenuates apoptosis and pyroptosis under OGD/R insult.** (**A**) The apoptosis rate of SH-SY5Y neurons as evaluated by flow cytometry with Annexin V/PI staining. (**B**) Expression of Pro-caspase 3 and Cle-caspase 3 in SH-SY5Y neurons as measured by western blot analysis. (**C**) Expression of NLRP3, ASC, caspase1, caspase8 and GSDMD in SH-SY5Y neurons as measured by western blot analysis. (**D**) The histogram of pyroptosis-associated proteins by western blot analysis. (OM-MSCs were replaced by MSCs in the figure. All data are presented as the mean value ± SD. *p<0.05, **p<0.01).

We investigated whether OGD/R could induce apoptosis in SH-SY5Y neurons. Results showed that cell apoptosis significantly increased after OGD/R injury. It was investigated whether IhOM-MSCs could reduce apoptotic cell death in SH-SY5Y neurons subjected to OGD/R injury. An OGD for 6 h and reperfusion for 24 h were the chosen conditions of the experiments. The results showed that IhOM-MSCs could significantly attenuate the rate of apoptosis in SH-SY5Y neurons relative to co-cultured cells with and without pretreated OM-MSCs after OGD for 6 h and reperfusion for 24 h (Figure 3A). In addition, a reduction in levels of caspase 3 was noted in SH-SY5Y neurons co-cultured with IhOM-MSCs relative to cells without pre-treatment (Figure 3B).

NLRP3 inflammasome activation and pyroptosis-associated proteins were measured in SH-SY5Y neurons after OGD/R injury. The results showed that NLRP3, ASC, caspase 1, caspase 8, and GSDMD proteins were significantly increased in SH-SY5Y neurons subjected to OGD/R injury (Figure 3C, 3D). The functional role of IhOM-MSCs was investigated on pyroptosis in SH-SY5Y neurons post-OGD/R injury. The results showed that NLRP3 inflammasome and pyroptosis-associated proteins were markedly decreased in SH-SY5Y neurons co-cultured with IhOM-MSCs (Figure 3C, 3D). This result was in comparison to cells with and without pre-treatment and given an miR-181a mimic after OGD for 6 h and reperfusion for 24 h.

**Figure 4:**
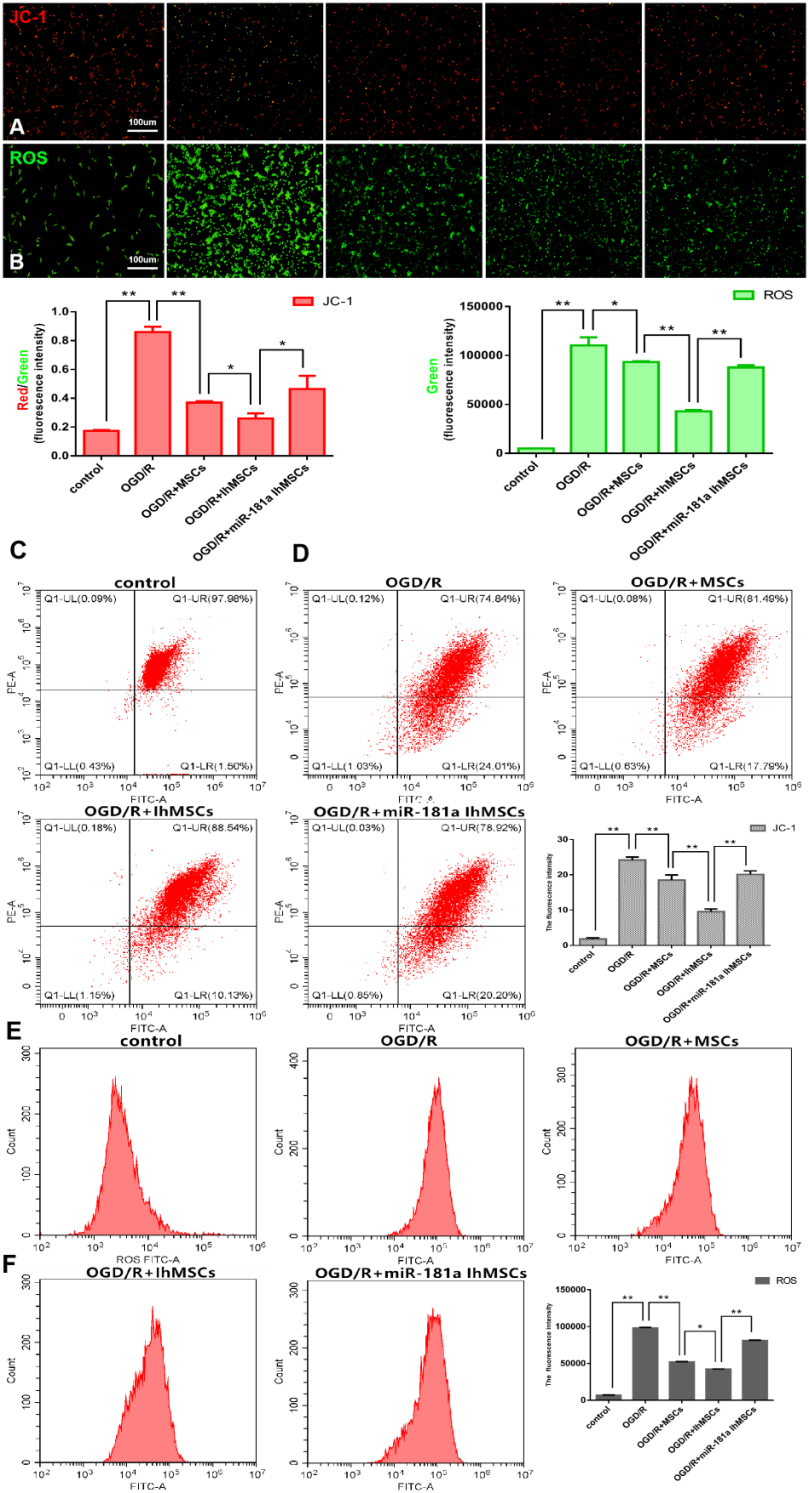
**Co-culture of IhOM-MSCs with SH-SY5Y neurons enhances the mitochondrial function recovery under OGD/R insult.** (**A**, **B**) Mitochondrial membrane potential (JC-1-labeled) and ROS were evaluated by immunofluorescence. (**C**, **D**) The histogram of JC-1 and ROS immunofluorescence. (**E**) Mitochondrial membrane potential was measured for using the JC-1-labeled by flow cytometry. (**F**) Levels of ROS were evaluated by flow cytometry. (OM-MSCs were replaced by MSCs in the figure. All data are presented as the mean value ± SD. *p<0.05, **p<0.01).

Mitochondrial function was measured for membrane potential using the labeled JC-1 and the levels of ROS. The results showed that IhOM-MSCs could increase MMP with a decrease of JC-1 green fluorescence (Figure 4A, 4C, 4E). In addition, there was a decrease in ROS levels in SH-SY5Y neurons subjected to OGD/R injury, relative to cells without pretreatment and an miR-181a mimic (Figure 4B, 4D, 4F).

### IhOM-MSC transplantation reduces the damaged area of the infarct cortex and improves motor function after ischemic stroke

**Figure 5:**
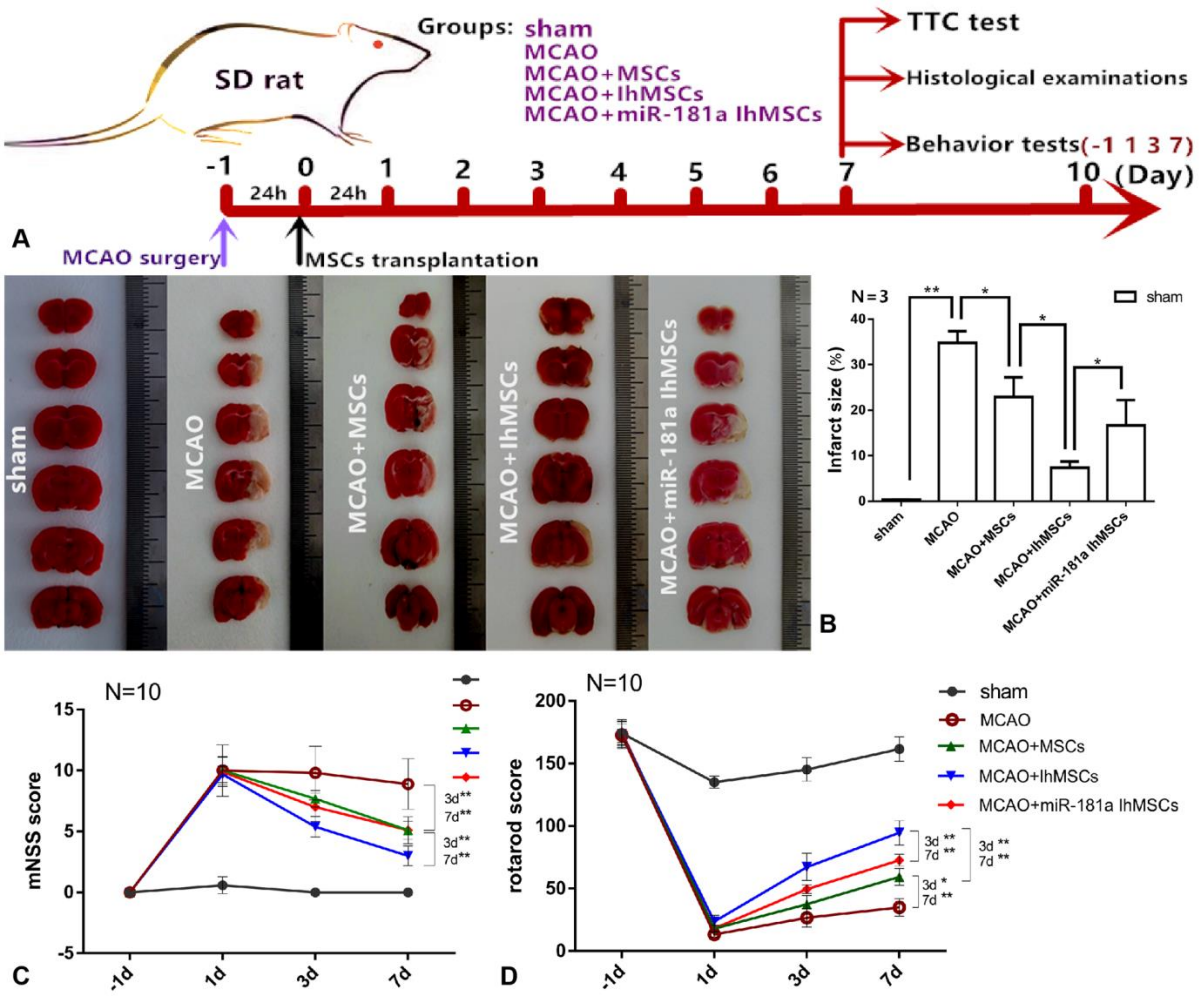
**IhOM-MSCs transplantation reduces the damaged area of the infarct cortex and improves motor function after ischemic stroke.** (**A**) The diagram shows the time course of the various experiments in this study. (**B**) TTC was used staining to determine the therapeutic effect of IhOM-MSCs on the infarct cortex. (**C**) The neurological deficits of rats were evaluated before and after cell transplantation using the mNSS scale. (**D**) The improvement of passive motor functions was evaluated in rats before and after cell transplantation by rota-rod treadmills. (OM-MSCs were replaced by MSCs in the figure. All data are presented as the mean value ± SD. *p<0.05, **p<0.01).

We were the first to investigate whether the OM-MSCs migrated to the infarction damaged area through the blood-brain barrier (BBB) after transplantation. Green fluorescent protein (GFP)-vector was transfected into OM-MSCs via injection of a lentivirus vector. Immunofluorescence results showed that there were GFP-positive OM-MSCs in the infarction area of SD rats, and the number of GFP-positive cells in the IhOM-MSCs group was significantly increased (Supplementary Figure 3). It was investigated whether MCAO surgery could cause cortical infarction. In addition, it was further researched whether IhOM-MSCs could reduce the damaged area of the infarct cortex in SD rats. MCAO for 2 h was chosen and MSC transplantation after reperfusion for 24 h was conducted. TTC staining and subsequent histological examination was performed on the 7th day after MSC transplantation. Behavior tests (including mNSS score and rotarod score) were performed after MSC transplantation at -1, 1, 3, and 7 days post-transplant (Figure 5A). TTC was used to determine the therapeutic effect of IhOM-MSCs on the infarct cortex. The damaged areas of the infarct cortices were markedly reduced in the IhOM-MSCs groups relative to those without pretreatment and an miR-181a mimic in MCAO rats (Figure 5B). Moreover, the neurological deficits of rats were evaluated before and after cell transplantation using the mNSS scale. The results showed that the mNSS scores of each group decreased over time. It was confirmed that OM-MSC transplantation improved the neurological function defects in rats (Figure 5C). The improvement in neurological function defects in the IhOM-MSCs transplantation group was significant compared with the other two groups (Figure 5C). The improvement of passive motor functions was evaluated in rats before and after cell transplantation by rota-rod treadmills. The results showed that the motor function in each group improved continuously over time (Figure 5D). The motor function of the IhOM-MSCs transplantation group improved significantly compared with the other two groups (Figure 5D).

### IhOM-MSCs transplantation attenuates apoptotic cell death and pyroptotic cell death after ischemic stroke

**Figure 6:**
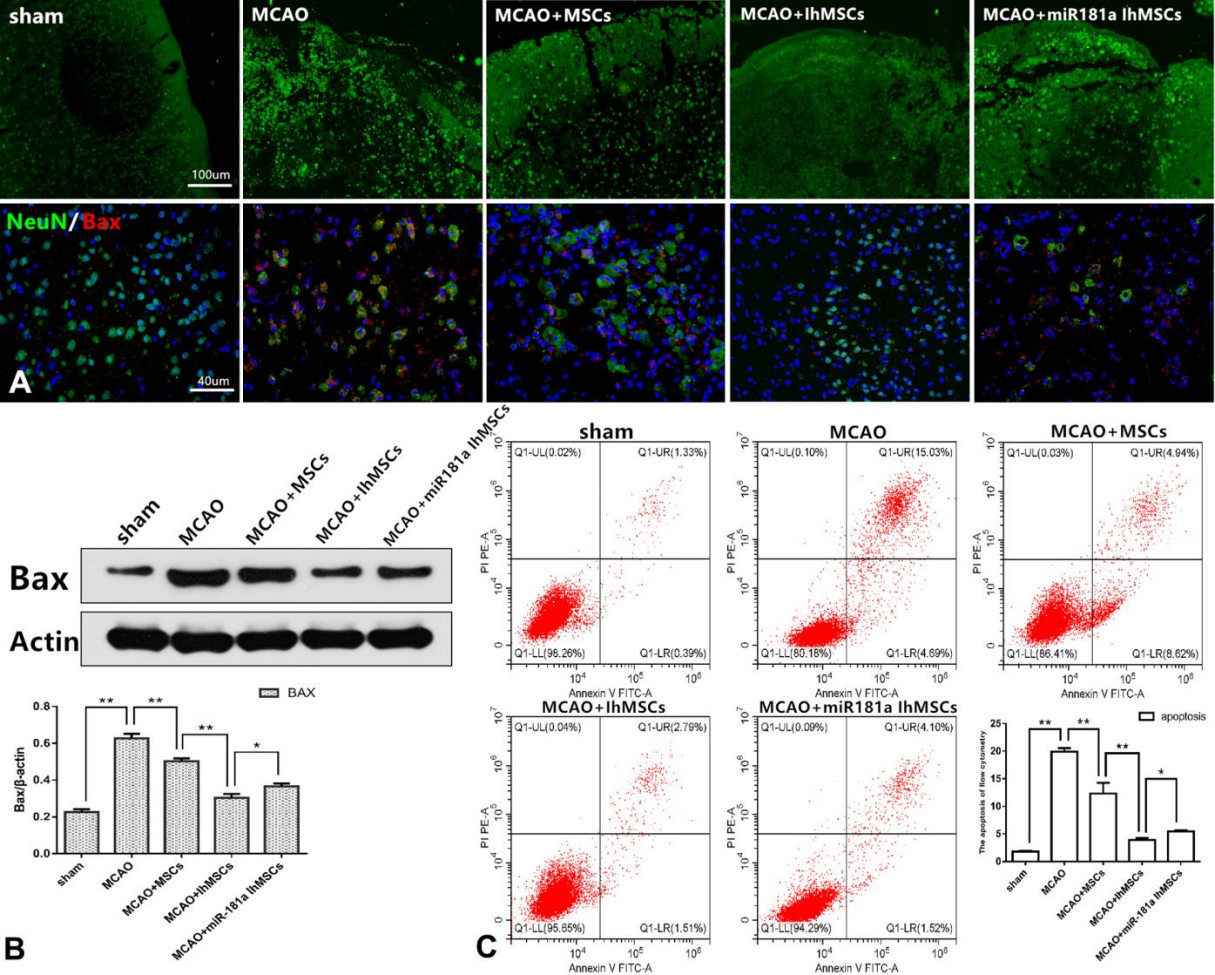
**IhOM-MSCs transplantation attenuates apoptotic cell death after ischemic stroke.** (**A**) Improvement in cortical tissue structure and apoptosis marker Bax were evaluated by immunofluorescence. (**B**) The apoptosis marker Bax of nerve cells was detected by western blot analysis. (**C**) The apoptosis rate of nerve cells as evaluated by flow cytometry with Annexin V/PI staining. (OM-MSCs were replaced by MSCs in the figure. All data are presented as the mean value ± SD. *p<0.05, **p<0.01).

It was investigated whether IhOM-MSCs could reduce apoptotic cell death in MCAO surgery rats. Apoptotic cell death was evaluated by immunofluorescence, western blot analysis and flow cytometry. Immunofluorescence results showed that the right cerebral cortex of MCAO rats was disorganized, discontinuous, and showed an irregular arrangement of nerve cells compared with the control group. It was confirmed that the infarcted cortical tissue structure was effectively restored in each group after cell transplantation. Improvement in tissue structure was most significant in the IhOM-MSCs group (Figure 6A). Immunofluorescence and western blot results revealed that the apoptosis marker Bax was significantly increased in rats with MCAO injury. Bax protein expression was observed to decrease in the IhOM-MSCs group (Figure 6A, 6B). Flow cytometry results showed that IhOM-MSCs attenuated the rate of apoptosis in nerve cells of the infarct cortex relative to those without pretreatment and an miR-181a mimic (Figure 6C).

**Figure 7:**
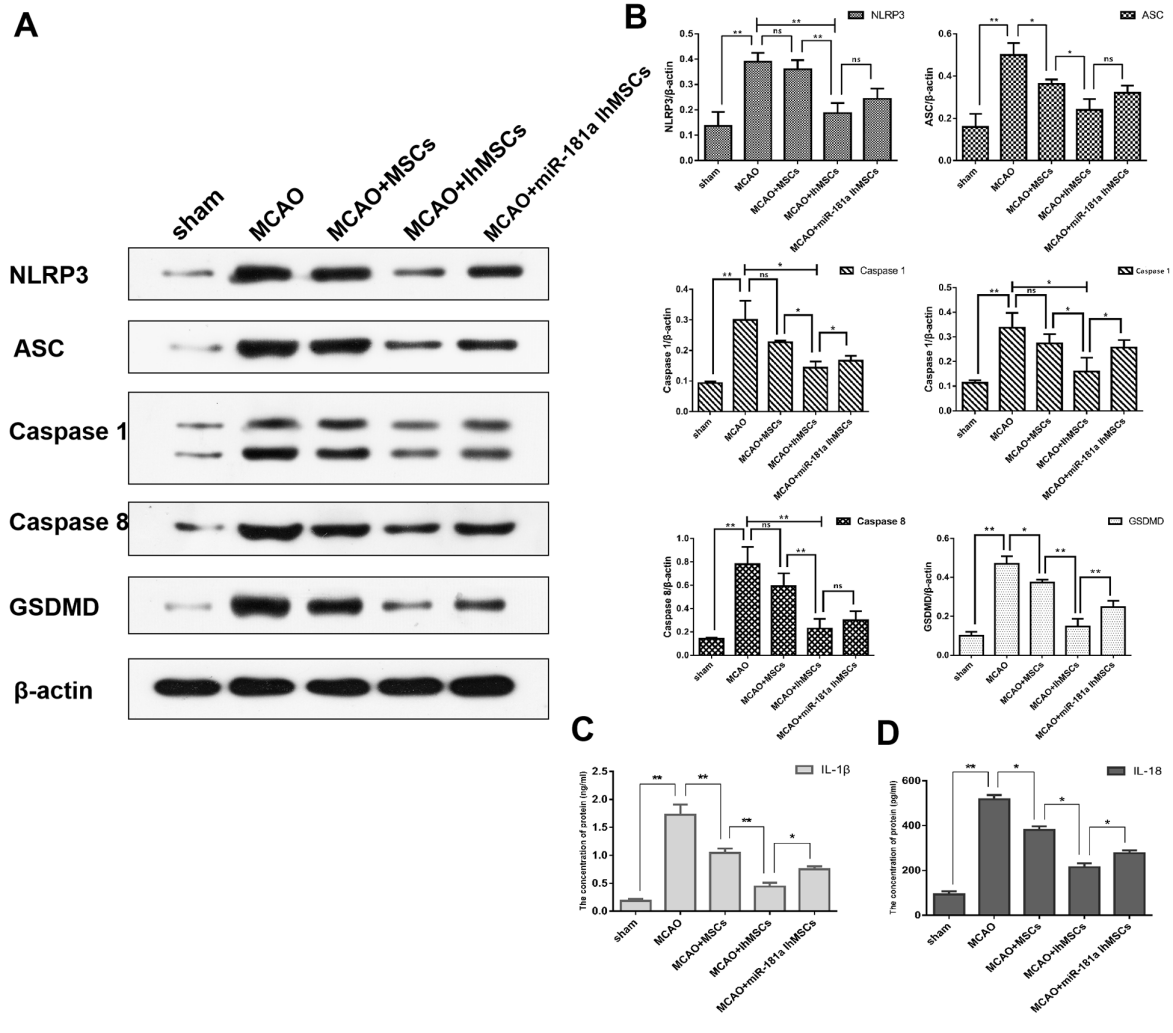
**IhOM-MSCs transplantation attenuates pyroptotic cell death after ischemic stroke.** (**A**) Expression of NLRP3, ASC, caspase1, caspase8 and GSDMD in nerve cells as measured by western blot analysis. (**B**) The histogram of western blot analysis including NLRP3, ASC, caspase1, caspase8 and GSDMD. (**C**, **D**) The content of IL-1β and IL-18 proteins were detected by ELISA assays. (OM-MSCs were replaced by MSCs in the figure. All data are presented as the mean value ± SD. *p<0.05, **p<0.01).

The functional role of IhOM-MSCs on nerve cell pyroptosis in MCAO surgery rats was then explored. Using western blot analysis, NLRP3 inflammasome activation was measured along with pyroptosis-associated proteins in MCAO surgery rats. The results showed that NLRP3, ASC, caspase 1, caspase 8 and GSDMD proteins were significantly increased in nerve cells of the infarcted cortex (Figure 7A, 7B). It was then investigated whether IhOM-MSCs could attenuate pyroptosis in nerve cells of the infarcted cortex. The data revealed that NLRP3 inflammasome and pyroptosis-associated proteins were markedly decreased in the IhOM-MSCs group relative to those without pretreatment and an miR-181a mimic in MCAO surgery rats (Figure 7A, 7B). Expression of IL-1β and IL-18 proteins were evaluated by ELISA assays. The results revealed that expressions of the IL-1β and IL-18 proteins increased in the cerebral infarction cortex of MCAO rats (Figure 7C, 7D). Expressions of the IL-1β and IL-18 proteins decreased in the IhOM-MSCs group, relative to cells without pretreatment and the miR-181a mimic (Figure 7C, 7D).

### IhOM-MSC transplantation enhances mitochondrial function recovery via miR-181a signaling after ischemic stroke

**Figure 8:**
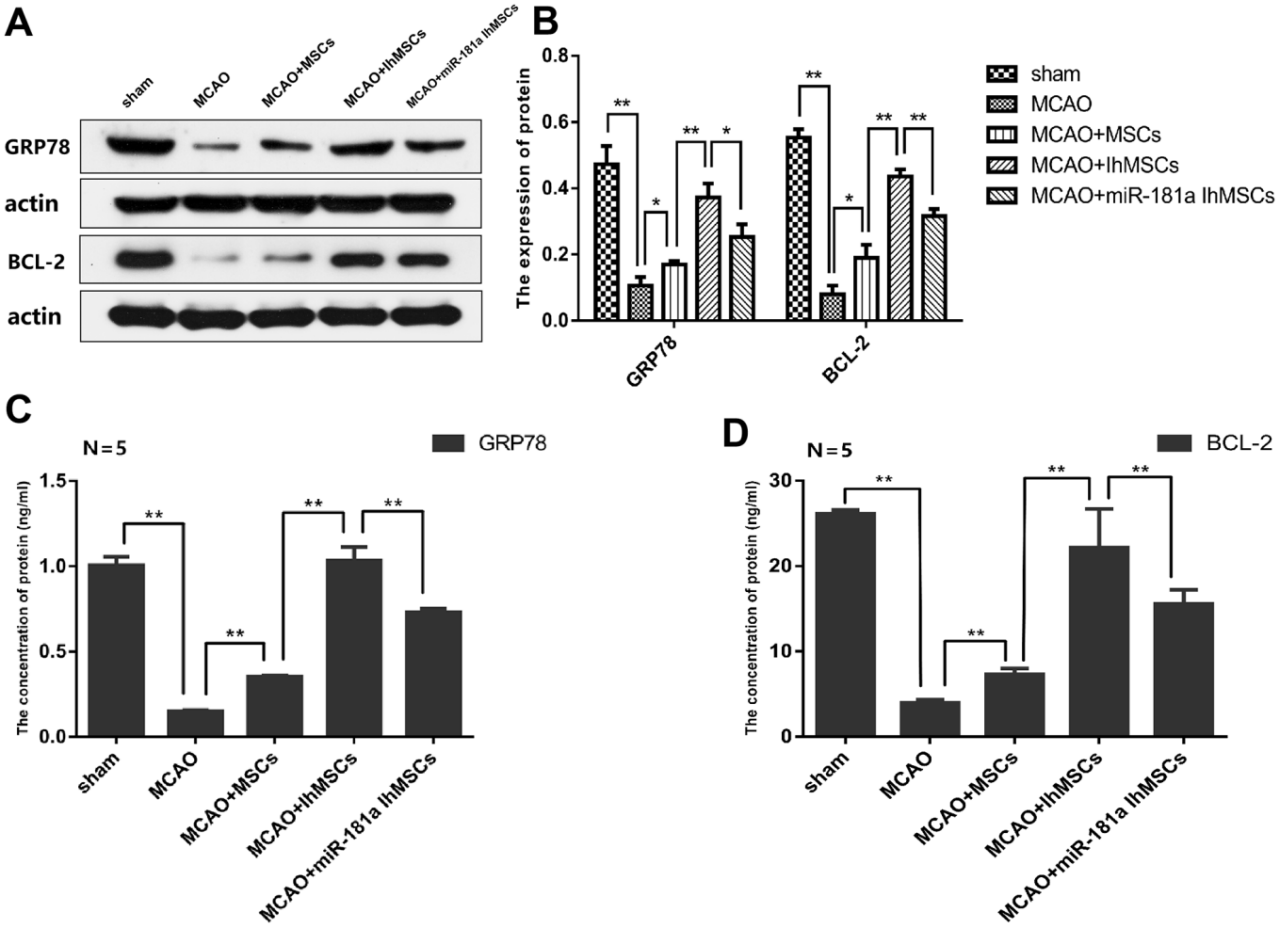
**IhOM-MSCs transplantation increased the expression levels of GRP78 and Bcl-2 proteins in infarcted cortical lesions.** (**A**, **B**) Expression of GRP78 and Bcl-2 proteins were evaluated by western blot analysis. (**C**, **D**) The content of GRP78 and Bcl-2 proteins were detected by ELISA assays. (OM-MSCs were replaced by MSCs in the figure. All data are presented as the mean value ± SD. *p<0.05, **p<0.01).

It was deduced whether IhOM-MSCs could protect neuron mitochondria and inhibit apoptosis and pyroptosis. The focus of this line of experimentation was on the expression of the miR-181a downstream target genes *GRP78* and *Bcl-2* in the cerebral infarction cortex of MCAO rats. Expression of GRP78 and Bcl-2 proteins were evaluated by western blot analysis and ELISA assays. Results revealed that expression of the GRP78 and Bcl-2 proteins decreased in the cerebral infarction cortex of MCAO rats (Figure 8A–8D). Expression of the GRP78 and Bcl-2 proteins increased in the IhOM-MSCs group, relative to cells without pretreatment and the miR-181a mimic (Figure 8A–8D).

**Figure 9:**
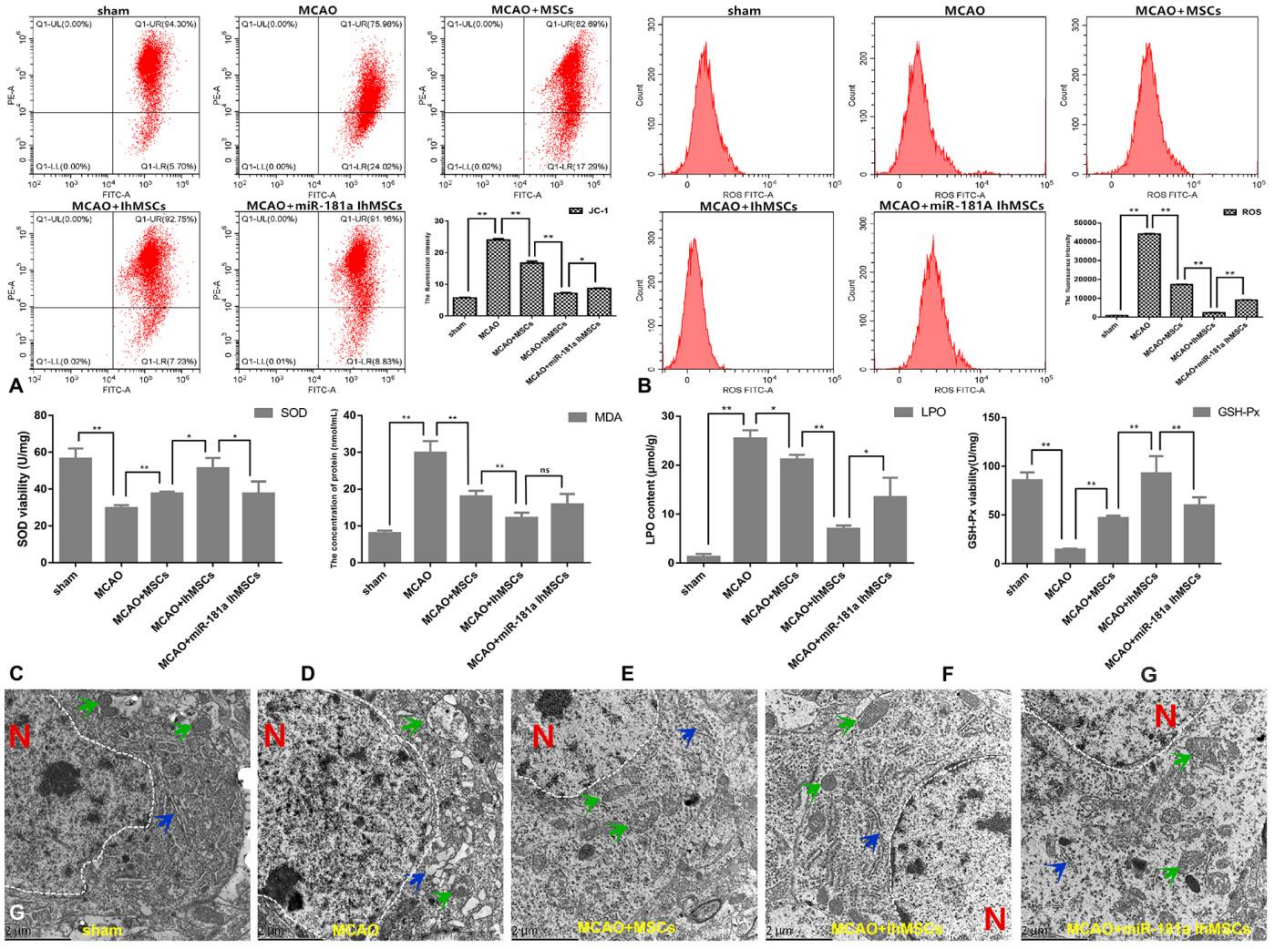
**IhOM-MSCs transplantation enhances the mitochondrial function recovery after ischemic stroke.** (**A**, **B**) Mitochondrial membrane potential was measured for using the JC-1-labeled by flow cytometry. (**B**) Levels of ROS were evaluated by flow cytometry. (**C**–**F**) To measure oxidative stress levels in cells, MDA/LPO contents and SOD/GSH-Px viabilities were measured by biochemical kit respective. (**G**) The mitochondrial and endoplasmic reticulum morphometric ultrastructural analyses were observed by transmission electron microscopy (TEM) in neurons of penumbra cortex. As shown in the figure: the white dotted lines represent the outlines of the nuclei, letter N represent nucleus, green arrows represent mitochondria and blue arrow represent endoplasmic reticulum. (OM-MSCs were replaced by MSCs in the figure. All data are presented as the mean value ± SD. *p<0.05, **p<0.01).

Mitochondrial function was measured for membrane potential using the labeled JC-1, and levels of ROS were assessed by flow cytometry. The results showed that IhOM-MSCs could markedly increase MMP by a noted decrease in the JC-1 green fluorescence (Figure 9A). Likewise, there was a decrease in ROS levels in IhOM-MSCs relative to those without pretreatment and the miR-181a mimic (Figure 9B). It was further investigated whether IhOM-MSCs had an antioxidative effect in MCAO surgery rats. Toward this end, SOD, MDA, LPO and GSH-Px were evaluated in the infarction cortex of MCAO rats using commercially available biochemical kits. The results from these experiments revealed that IhOM-MSCs increased antioxidant response elements (SOD and GSH-Px) and decreased oxidation reaction elements (MDA and LPO) relative to those without pretreatment and the miR-181a mimic (Figure 9C–9F).

Finally, it was investigated whether MCAO surgery could cause organelle structural disorder. Likewise, it was determined whether IhOM-MSCs could markedly reduce organelle structural disorder in neurons of the infarcted cortex. Transmission electron microscopy was used to analyze the ultrastructure of mitochondria and endoplasmic reticulum organelles in these neurons (Figure 9G). In this figure, the white dotted lines represent the outlines of the nuclei, the letter N represents the nucleus, the green arrows represent the mitochondria, and the blue arrows represent the endoplasmic reticulum. Our analysis showed that the mitochondria and endoplasmic reticulum became disorganized in MCAO rats. For example, organelles appeared swollen, dilated, and fractured compared with the control group (Figure 9G). It was confirmed that the organelle structural disorder was effectively restored in each group after cell transplantation, where improvement was most prominent in the IhOM-MSCs group (Figure 9G).

## DISCUSSION

MSCs are adult stem cells which are widely distributed in tissues and organs. MSCs not only have the potential of multidirectional differentiation but also have multiple functions such as self-replication, paracrine function, and immune regulation [11, 18]. MSCs can be used for tissue repair, organ regeneration, and gene therapy to resolve many medical problems. Currently, there are three kinds of MSCs that have been widely studied. These include bone marrow MSCs, adipose-derived MSCs, and umbilical cord MSCs. In 2010, researchers found that OM-MSCs isolated from human nasal mucosa were the ideal source for MSCs [19]. These cells are also known as ectomesenchymal stem cells because they are derived from embryonic ectoderm [20]. OM-MSCs as a new type of MSCs not only have the general biological characteristics of other MSCs but have many additional advantages. These cells have a higher proliferation efficiency and a shorter passage time. The cells are widely distributed in the nasal cavity, are easily accessible, show no immune rejection and have little or no associated ethical issues [11, 21]. The OM is regenerative tissue and can be renewed for life. It has the added benefit of retaining biological activity and thus does not change with age [13]. As stated earlier, nasal mucosa originates in the embryonic ectoderm and can directly differentiate into neurons under the appropriate conditions. Chromosome karyotype analysis and tumor gene analysis have shown that there is no genetic variation after substantive *in vitro* passages [12]. Our results confirmed that OM-MSCs had a multidirectional differentiation potential that included transformation to neurons, bone tissue, and adipose tissue. Thus, the advantages of OM-MSCs make it an ideal source of seed cells for the treatment of ischemic stroke.

Although MSCs in the treatment of ischemic stroke have great potential, there remain substantial problems to solve. One of the most important issues is that in an ischemia reperfusion lesion area, there is a shortage of blood supply and a large number of free radicals. This combination of effects can lead to impaired activity and decreased paracrine function of the transplanted MSCs, thus the therapeutic effect can be affected [22, 23]. To overcome these problems and improve their therapeutic effect on ischemic stroke, new and innovative research approaches have emerged to optimize culture conditions, transplantation pathways, and cell number during the process of MSC transplantation. We hypothesized that optimization by changing the culture conditions could potentially play a synergistic role in the treatment of ischemic stroke. Studies have demonstrated that hypoxic preconditioning (2%–5% O_2_) can maintain the homogeneity and differentiation potential of MSCs, and delay cell senescence while increasing the expression of MSC chemokine receptors [24–26]. Hypoxic preconditioning can also increase the paracrine function of MSCs, thus enhancing their effectiveness in the treatment of ischemic stroke, myocardial infarction, and posterior limb ischemia [27–29]. We previously demonstrated that after ischemic and hypoxic preconditioning of OM-MSCs, the expression of a series of paracrine factors was changed, such as vascular endothelial growth factor, nerve growth factor, brain derived neurotrophic factor, glial cell line-derived neurotrophic factor and matrix metalloproteinase-2, all of which play an important role in the biological functions of MSCs. In the present study, it was hypothesized that ischemic-hypoxic preconditioning of OM-MSCs (IhOM-MSCs) *in vitro* could simulate the ischemia/reperfusion microenvironment in ischemic stroke. In doing so, enhancing the adaptability and paracrine ability of transplanted cells might arrive at a better therapeutic effect. We also determined whether the OM-MSCs could migrate to the infarction damaged area through the BBB after transplantation. Furthermore, we determined changes in the migration ability of OM-MSCs after ischemic-hypoxic preconditioning. Studies have shown that the BBB is impaired and permeability was significantly increased in animals and patients with ischemic stroke [30, 31]. Our results showed that OM-MSCs migrated to the infarction area through the BBB, and that the migration ability of IhOM-MSCs was improved. This indicated that ischemic-hypoxic preconditioning improved the homing ability of OM-MSCs, although the specific mechanism remains to be further studied. Our results also showed that IhOM-MSC transplantation reduced the damaged area of the infarct cortex, thereby improving motor function after ischemic stroke.

Cerebral ischemia/reperfusion injury causes pathological processes in the brain that involves complex temporal and spatial cascades. Mitochondria play an important role in the energy metabolism of the brain and the regulation of Ca2+ balance in cells. Mitochondria are also the primary contributor to the production of ROS [32, 33]. The process of apoptosis triggered by neuronal injury in cerebral ischemia has been shown to involve mitochondrial dysfunction [34, 35]. The mitochondrial dysfunction involves an inability of oxidative phosphorylation to carry out ATP production effectively. The result is cellular dysfunction due to insufficient energy [36, 37]. Furthermore, with prolongation of ischemia, mitochondrial function and structure are damaged and ATP depletion leads to further damage of cellular functions [38]. When cerebral ischemia progresses to the reperfusion stage, mitochondria tend to produce a large number of free radicals, with an accompanying increase in calcium transport disorders. This results in an irreversible permeability change in the mitochondrial membrane and a sharp increase in cytoplasmic calcium [39]. Experiments have shown that apoptosis is induced by apoptosis-related factors such as those in the b-cell combat-2 (Bcl-2) family, caspase, and cytochrome-c (Cyt-c) [40, 41]. Our data showed that IhOM-MSCs attenuated apoptosis of nerve cells relative to OM-MSCs without pretreatment after ischemic stroke. In addition to these factors, NLRP3 inflammasomes played an important role in pyroptosis involving the mitochondria, such that NLRP3 activators could induce mitochondrial destabilization. NLRP3 activators have likewise been shown to induce NLRP3 deubiquitination and the release of mitochondria-derived molecules such as cardiolipin and mitochondrial DNA [42, 43]. These molecules bind to and activate the NLRP3 inflammasome, which is transferred to the mitochondria. The studies have also confirmed that NLRP3 inflammasome activation can be regulated by mitochondrial ROS and MMP [42, 44, 45]. Our data showed that IhOM-MSCs attenuated pyroptosis of nerve cells relative to OM-MSCs without pretreatment after ischemic stroke. It is particularly important to protect the mitochondrial function of nerve cells when ischemic stroke occurs. For this purpose, we changed the culture conditions of OM-MSCs *in vitro* to optimally increase protective mitochondrial function. Our results showed that IhOM-MSCs inhibited the production of ROS and increased the membrane potential of mitochondria, when compared with OM-MSCs. The intervention by IhOM-MSC transplantation could markedly enhance the mitochondrial functional recovery and reduce the oxidative stress response in neurons of infarcted cortex after ischemic stroke. Changes of miRNAs in ischemic stroke have been detected by miRNA amplification techniques such as the mouse middle artery embolization model (MCAO). This suggests that miRNAs are potential biomarkers and possible therapeutic targets for stroke [46, 47]. The miR-181 family is evolutionarily conserved and highly expressed in the brain. It has been closely associated with apoptosis and inflammation [48]. Studies have shown that miR-181a is significantly downregulated in stroke models and has protective effects on neurons and glial cells [16, 17]. In this study, IhOM-MSCs by simulating the microenvironment in stroke pathology. Optimized IhOM-MSCs were better able to tolerate ischemic stroke injury, thus increasing cellular activity. Likewise, the downregulation of miR-181a in IhOM-MSCs increased the function of protecting the mitochondria of nerve cells in the ischemic lesion area.

The miR-181a can target and regulate two downstream protein families: heat shock protein 70 (HSP70), glucose regulated protein 78 (GRP78), anti-apoptotic Bcl-2, and myeloid cell leukemia-1 (McL-1) [16, 49]. Both the HSP70 and Bcl-2 families can affect mitochondrial function and protect neurons from ischemia when overexpressed. In this study, we confirmed the upregulation of the downstream target genes, *GRP78* and *Bcl-2*, by miR-181a downregulation of IhOM-MSCs, when compared with OM-MSCs that were not pretreated. Researchers have demonstrated that miR-181a antagonists can exert a neuroprotective effect on the damaged area in the rat MCAO model by upregulating the downstream target gene GRP78 [49]. The GRP78 protein was originally thought to be found in the endoplasmic reticulum, but recent studies have shown that GRP78 can target multiple organelles such as mitochondria in different cell types [50]. During the UPR phase of endoplasmic reticulum stress, GRP78 localization to the mitochondria reduced the calcium ions in the mitochondria increased by endoplasmic reticulum stress. This, in turn, inhibited the reduction of MMP induced by endoplasmic reticulum stress, thereby reducing the damage to mitochondrial function in cells [51]. The miR-181a also induces mitochondrial dysfunction and increases apoptosis in primary astrocytes by regulating the target genes, *Bcl-2* and *Mcl-1,* downstream [16, 52]. The miR-181a also induces mitochondrial dysfunction and increases apoptosis in primary astrocytes by regulating the target genes, *Bcl-2* and *Mcl-1*, downstream [52]. *Bcl-2* is a representative repressor of apoptosis, which is distributed on the cytoplasmic surface of the mitochondrial outer membrane. *Bcl-2* stabilizes the function of the mitochondrial membrane and can inhibit the release of Cyt-c and active factor AIF [52]. *Bcl-2* can also act on the related proteins of MPT to prevent the opening of mPTP and maintain the transmembrane potential of the mitochondrial membrane. This serves to decrease ROS production and protect mitochondrial function [53]. In the present study, it was demonstrated that the two target proteins, GRP78 and Bcl-2, were significantly upregulated in the lesion area after transplantation of IhOM-MSCs to the MCAO rat model compared with OM-MSCs that were not pretreated. IhOM-MSCs have a greater capacity to protect the mitochondria in nerve cells in the lesion area, thereby reducing both apoptosis and pyroptosis of neurons that promote neurogenesis and neuroprotection.

In conclusion, the present study demonstrated the efficacy of OM-MSC transplantation in the treatment of ischemic stroke in rats with an increased benefit using IhOM-MSCs. The search for better seed cell sources and cell optimization programs is a trend in the treatment of refractory diseases. Advantages of using OM-MSCs include: abundant source material, high level of safety, autologous transplantation avoiding immune rejection, and no ethical issues. Thus, IhOM-MSCs are a promising and reliable source for future ischemic stroke therapies and other refractory diseases.

## MATERIALS AND METHODS

### Ethics statement

Sprague-Dawley (SD) rats obtained from the laboratory animal department of Central South University were used in the study. This study was conducted in strict accordance with the guidelines established by the Committee on the Use and Care of Animals at the Hunan Province, China. All efforts were made to minimize pain. The protocol was approved by the Ethics Committee of the University of Hunan Normal University (Number: 2020-164).

### Preparation of OM-MSCs and IhOM-MSCs

Prior to harvesting OM, volunteers signed informed consent forms and were evaluated for hematology, blood biochemistry and urine microbiology. Volunteers were also screened for infectious diseases. OM were thrice rinsed in DMEM/F12 (v/v=1:1) (Gibco, Grand Island, NY, USA) mixed medium containing penicillin (200 U/mL) and streptomycin (200 U/mL) to remove blood, and then were placed in DMEM/F12 medium containing 10% fetal bovine serum (FBS; HyClone, Logan, UT, USA), 100 U/mL penicillin, and 100 U/mL streptomycin. They were then minced with ophthalmic scissors into 1 mm^3^ pieces, which were then centrifuged to remove the supernatant. Pellets were seeded in Corning culture flasks (Corning, Corning, NY, USA). When the cell confluence reached 100%, the cells were trypsinized and passaged. OM-MSCs at passage four were used for the assays, including immunofluorescence, flow cytometry, electron microscopy, and multidirectional differentiation potential. To prepare IhOM-MSCs, OM-MSCs were pretreated with ischemia-hypoxia (5% FBS + 3% O_2_).

### OGD/R neurons and the MCAO rat model

Procedures for the OGD/R neuron model: the neuron (SH-SY5Y) maintenance medium was replaced with Dulbecco's Modified Eagle’s Medium (Gibco, USA). The cells were then cultured in a three gas incubator (37° C, 95% N_2_, 5% CO_2_) for 6 h. Thus, the hypoxia and hypoglycemia environment of cultured neurons was created to simulate the situation of cerebral ischemia *in vivo*. The cells were rinsed three times with phosphate-buffered saline (PBS), then incubated in DMEM/F12 containing 10% FBS (37° C, 21% O_2_, 74% N_2_, 5% CO_2_) for the purpose of ischemia reperfusion. Transwell co-culture plates (Corning, USA) were used to co-culture SH-SY5Y cells and OM-MSCs. The OM-MSCs were placed in the upper compartment and SH-SY5Y cells were placed in the lower compartment for 24 h.

Procedures for the MCAO rat model: Adult Sprague-Dawley rats (weighing 250–300 g) were anesthetized with chloral hydrate (0.35 mL/100 g, intraperitoneally). The rats were placed in a supine position after successful anesthesia. Rubber strips were used to secure the rat's limbs and head. Fur from the surgical area of the neck was cleared and iodine was used to disinfect the skin for surgery. A longitudinal incision (1.5–2 cm) was made in the middle of the neck. The right common carotid artery (CCA) and its branches, as well as the external carotid artery and the internal carotid artery (ICA) were exposed after subcutaneous fascia and muscle separation. The CCA proximal end and the ECA bifurcation were ligated with surgical sutures and the ICA was closed with an arterial clip. An incision was cut in the middle of the CCA and a fishing line was then inserted from the CCA incision into the ICA. The ICA artery clip was loosened and the line was continued up into the intracranial segment. The plug line, of approximately 10 mm, was blocked for 2 h, then slowly and gently removed. After awaking from anesthesia, the model rats were given behavioral scores according to Zea Longa's 5-point assessment criteria; the rats with scores of 1–3 were rated as successful models and included in the study. Any rats with scores of 0 and 4, as well as expired rats, were excluded.

### Transplantation of OM-MSCs

The rats were placed in a bucket fixator, with the tail naturally extended and exposed to the outside. There was a vent hole at the end of the fixator for the rats to breath, and the tail was immersed in warm water for 2 min. The veins were wiped with 75% alcohol to promote dilation and the softening of epidermal cutin, after which the tails were tied with a rubber band to facilitate further filling of the veins. Finally, cells were injected into the tail (a 1/3 of the way down the tail) after disinfection with iodine complex. The number of cell transplants was approximately 1 × 10^6^. The cells were suspended in 0.5 ml and slowly injected into the rat tail vein at a rate of 0.1 mL/min.

### Measurement of infarct sizes

Rats (n=3/group) were deeply anesthetized using 10% chloral hydrate, then decapitated. Brain tissue was washed with 0.9% saline to remove blood stains, then the tissue was placed in a refrigerator for 30 min at −20° C. Coronal sections were prepared using 2-mm thick sections. The prepared slices were soaked in 1% 2,3,5-triphenyltetrazolium chloride TTC; Wellbio, Shang Hai, China), and fixed in 4% paraformaldehyde for 10 min. The size of the infarct area without red staining was determined from digital images using ImagePro software (Media Cybernetics, Rockville, MD, USA).

### Immunofluorescence

Rats were anesthetized and perfused transcardially with 4% paraformaldehyde. Frozen sections were cut using a cryostat. Immunofluorescence was performed using standard protocols. Briefly, after fixation and washing, the cultures were blocked with 10% normal goat serum in 0.3% Triton X-100 (Sigma-Aldrich, St. Louis, MO, USA) for 1 h at room temperature, then incubated with the primary antibody overnight at 4° C. The cultures were then incubated with fluorescence-conjugated secondary antibodies for 1 h at room temperature, and mounted with a coverslip and medium containing 4',6-diamidino-2-phenylindole (DAPI) (Beyotime, Hangzhou, China) to counterstain the nuclei. Images were captured using a digital camera and fluorescence microscope (Carl Zeiss Axioskop2+, Jena, Germany).

### Quantitative real-time PCR

The total RNA was extracted from cells using the acid guanidinium isothiocyanate-phenol-chloroform method with TRIzol reagent (Sigma-Aldrich) and reverse-transcribed for cDNA synthesis using a SuperScript III cDNA synthesis kit (Sigma-Aldrich). Each cDNA subpopulation was subjected to PCR amplification using specific primers. The PCR products were mixed with a loading buffer (0.25% Bromophenol Blue, 0.25% xylene cyanol, and 40% sucrose) (Sigma-Aldrich) and separated on 2% agarose gels. The data were analyzed using MxPro Q-PCR software.

### Western blotting

Cells and brain tissue were dissolved with sodium dodecyl sulfate (SDS) (Amresco, Solon, OH, USA) buffer (62.5 mM Tris-HCl, 10% glycerol, 2% SDS, and 50 mM dithiothreitol). The proteins were then transferred to polyvinylidene difluoride (PVDF) (Amresco, Solon, OH, USA) membranes. The blots were blocked in 4% bovine serum albumin (BSA) (Amresco, Solon, OH, USA) in Tris-buffered saline tween-20 (TBST) (Amresco, Solon, OH, USA) solution for 30 min at room temperature and then incubated at 4° C overnight with primary antibodies. After incubation with secondary antibodies at room temperature for 1 h, the blot was visualized using the Chemi Doc XRS imaging system (Bio-Rad, Hercules, CA, USA).

### Biochemical determinations

The levels of protective mitochondria cytokines (GRP78, Bcl-2) and proinflammation cytokines (IL-1β, IL-18) were detected using ELISA kits (Beyotime, Shang Hai, China) according to the recommended protocols. The absorbances were measured at 450 nm using a microplate reader (Thermo Fisher Scientific, Waltham, MA, USA). To measure oxidative stress levels in cells, we measured the malondialdehyde/lipid peroxide (MDA)/(LPO) contents and superoxide dismutase/glutathione peroxidase (SOD)/(GSH-Px) viability using a biochemical kit (Beyotime, Shang Hai, China) according to the recommended protocols.

### Scanning and transmission electron microscopy

The surface morphology of OM-MSCs was observed by scanning electron microscopy, and the mitochondrial ultrastructure of neurons of the penumbra cortex was observed by transmission electron microscopy. To evaluate the surface morphology of OM-MSCs, fresh cell samples were fixed in 2.5% glutaraldehyde for 1 h at room temperature, washed in phosphate buffer solution three times, dehydrated with ethanol, and then dried for 12 h. Finally, double-sided adhesive tape was used to paste the sample onto the scanning electron microscope copper plate. To evaluate the ultrastructure of mitochondria, fresh tissue samples were fixed in 2.5% glutaraldehyde overnight at 4° C, washed in cacodylate buffer, and post-fixed in 1% osmium tetroxide. Ultrathin sections of embedded tissues were stained with 5% uranyl acetate and lead citrate solution. The surface morphology of OM-MSCs and the mitochondrial ultrastructure of neurons were observed using an electron microscope (HT7700, Hitachi, Tokyo, Japan).

### Behavioral tests

Two behavioral tests were performed (n=12/group), with the highest and lowest values discarded. A modified neurological severity score (mNSS) test evaluated the degree of neurological impairment in MCAO rats, including motor, sensory, reflex, epilepsy, and myoclonic, as well as dystonia. A rotarod test evaluated the coordinated limb movements by measuring the latency at which the rats remained on a slowly accelerated rotating pole. The parameters of the rotarod treadmills were set as follows during the experiment: total time (300 s), acceleration time (20 s), maximum rotation speed (20 rotations), minimum rotation speed (3 rotations), and touch electricity time (3 s).

### Statistical analysis

Statistical analyses were performed using GraphPad Prism 8 software (GraphPad Software Inc., San Diego, CA, USA). Measurement data are presented as means ± SEM. Comparisons among groups were performed using the *t*-test or analysis of variance (ANOVA). A statistically significant difference was defined as ^*^P<0.05 and ^**^P<0.01.

## Supplementary Material

Supplementary Figures
