# Supplementary material for: Ischemic-hypoxic preconditioning enhances the mitochondrial function recovery of transplanted olfactory mucosa mesenchymal stem cells via miR-181a signaling in ischemic stroke
Source: Aging (Albany NY). 2021 Apr 4;13(8):11234–56. doi: 10.18632/aging.202807 (PMC8109091; doi:10.18632/aging.202807)
Supplement: Supplementary Figures [file aging-13-202807-s001.pdf]

SUPPLEMENTARY FIGURES

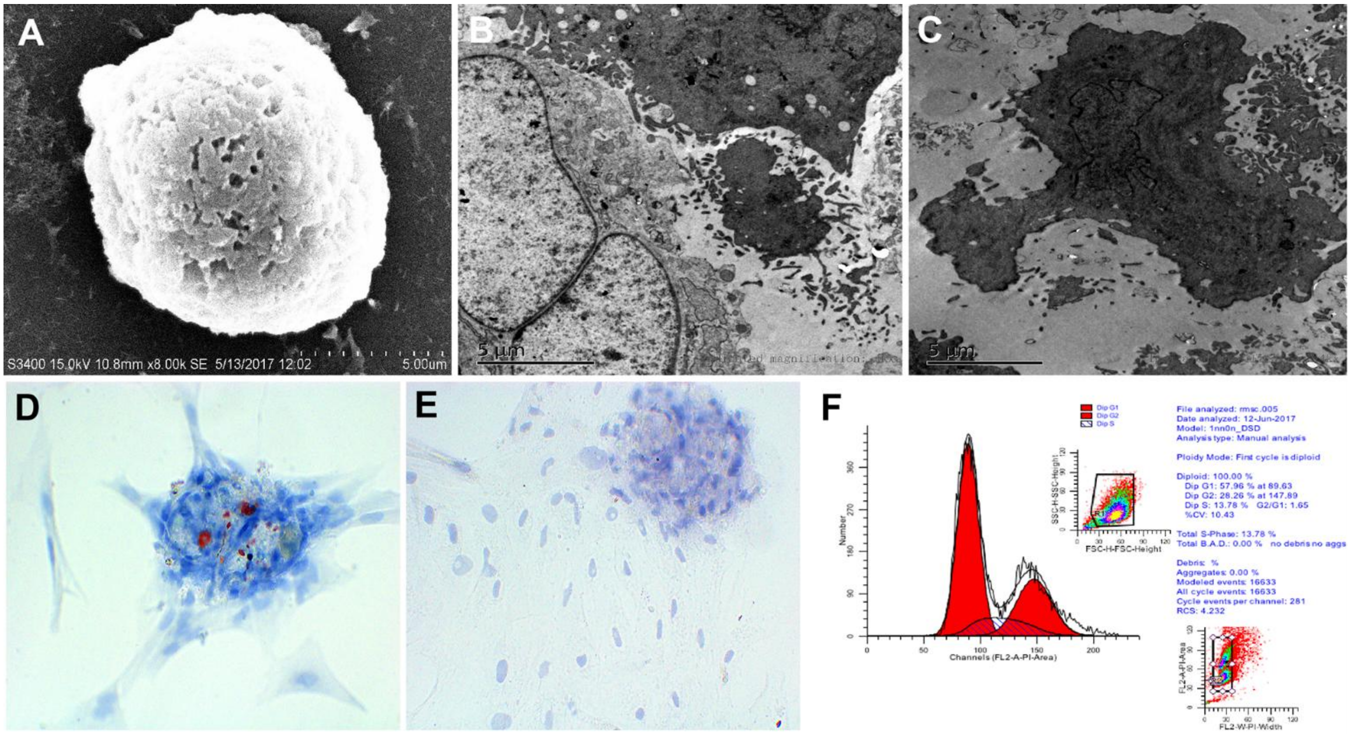

Supplementary Figure 1. Biological characteristics of OM-MSCs. (**A**) The surface of OM-MSCs were observed by scanning electron microscope. (**B**, **C**) Ultrastructure of the cells were observed by transmission electron microscopy (TEM). (**D**) OM-MSCs induced by a lipogenic inducer were stained with Oil Red O. (**E**) OM-MSCs induced by an osteogenic inducer were stained with alizarin red. (**F**) The cell cycle detected by flow cytometry.

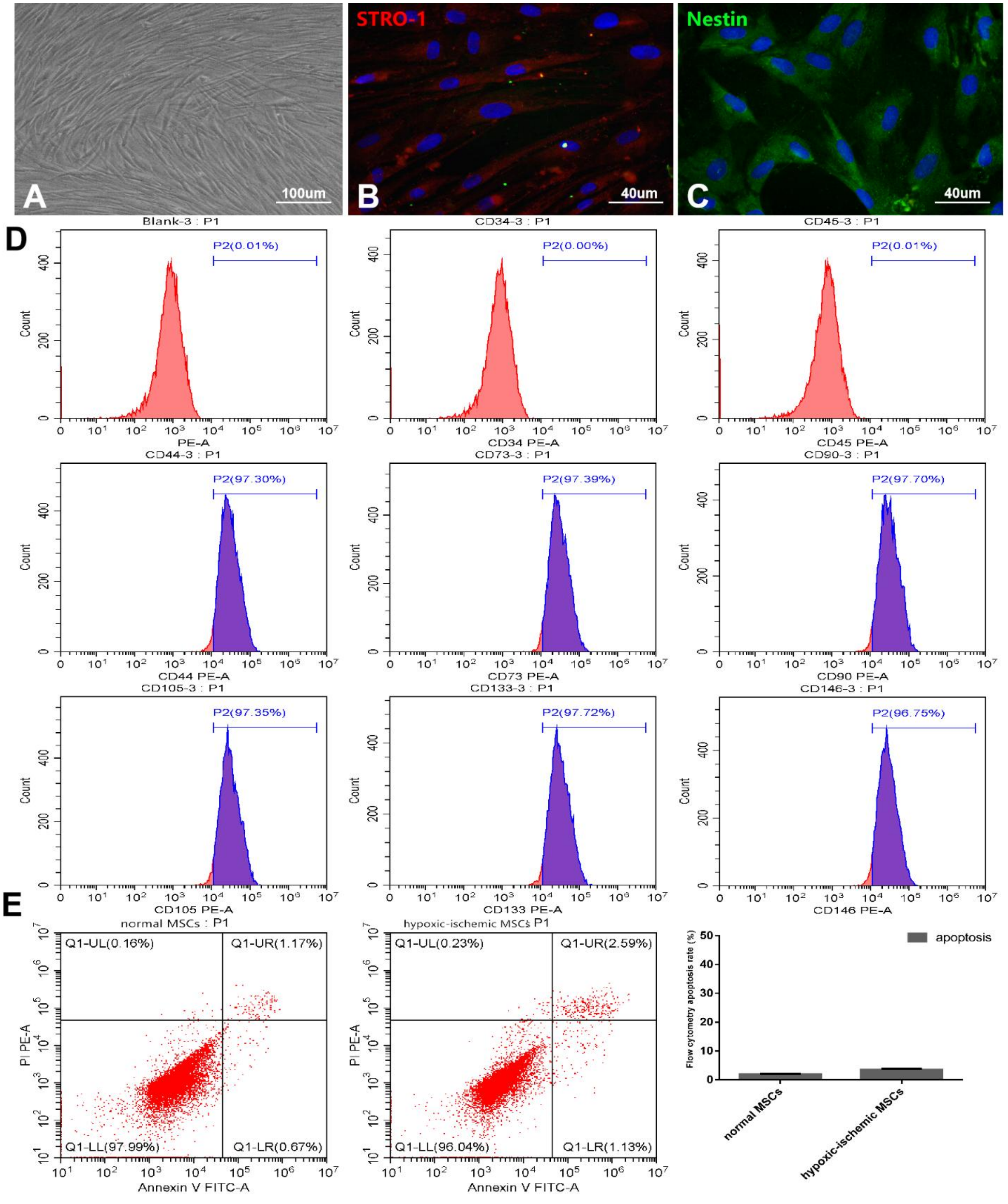

Supplementary Figure 2. Cell specific markers and apoptosis rates were compared between IhOM-MSCs and OM-MSCs. (**A**) IhOM-MSCs were mainly exhibited spindle-shaped and a radial arrangement under light microscope. (**B**, **C**) The specific markers STRO-1 and Nestin of IhOM-MSCs were identified by immunofluorescence. (**D**) The surface markers and purity of IhOM-MSCs were detected by flow cytometry assay. (**E**) The apoptosis rate of both cells as evaluated by flow cytometry with Annexin V/PI staining.

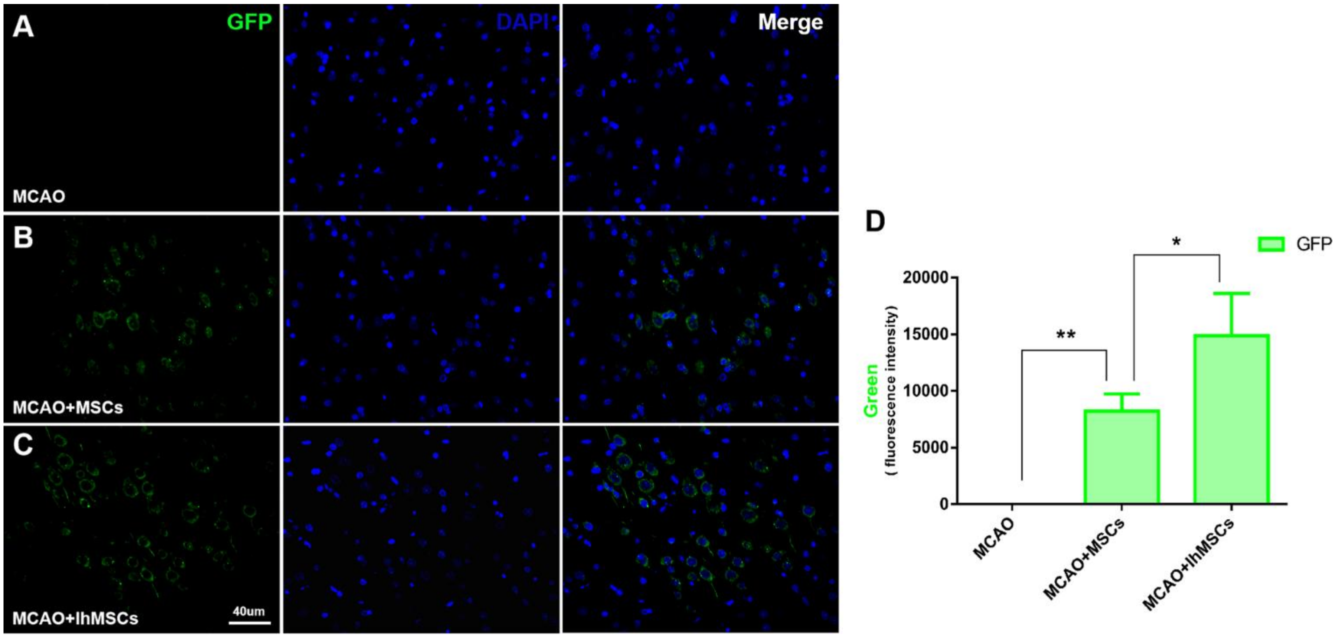

Supplementary Figure 3. NEW-OM-MSCs could migrate to the infarction area through the blood-brain barrier after transplantation. (**A**) No GFP-positive cells were found in MCAO group. (**B**) GFP-positive cells were found in MCAO+MSCs group. (**C**) The number of GFP-positive cells was significantly increased in the MCAO+IhMSCs group compared with the MCAO+MSCs. (**D**) Histogram of the number of GFP positive cells in each group. (OM-MSCs were replaced by MSCs in the figure. All data are presented as the mean value ± SD. *p<0.05, **p<0.01).
